# Supplementary material for: The Bibliometric Landscape of Gene Editing Innovation and Regulation in the Worldwide
Source: Cells. 2022 Aug 29;11(17):2682. doi: 10.3390/cells11172682 (PMC9454589; doi:10.3390/cells11172682)
Supplement: Supplementary file 1 [file cells-11-02682-s001.zip › cells-1812254-supplementary.pdf]

The Bibliometric Landscape of Gene Editing Innovation and Regulation in the Worldwide

Xun Wei [1,2,*], Aqing Pu [1], Qianqian Liu [1], Quancan Hou [1,2], Yong Zhang [2], Xueli An[1,2], Yan Long[1,2], Yilin Jiang [1], Zhenying Dong[2], Suowei Wu [1,2] and Xiangyuan Wan [1,2,*]

[1]Zhongzhi International Institute of Agricultural Biosciences, Research Center of Biology and Agriculture, Shunde Graduate School, University of Science and Technology Beijing, Beijing 100024, China
[2]Beijing Beike Institute of Precision Medicine and Health Technology, Beijing 100192, China
*Correspondence: weixun@ustb.edu.cn (X.Wei); wanxiangyuan@ustb.edu.cn (X.Wan); Tel.: +86-189-1087-6260 (X.Wei); +86-186-0056-1850 (X.Wan)

Materials and Methods

Records are retrieved and cross-indexed by using entries providing information with regards to manuscript authors, affiliated institutions, publication journals, years, titles and abstracts, spanning manuscripts published between 1999 and 2022 (up to January 19, 2022). The search strategy is based on the gene- editing- related queries (Supplementary Table 1). The literature types are limited to articles and reviews written in English.

Data cleaning is conducted within the retrieved literature to eliminate meetings, newspapers and unrelated literature, and finally streamlined the results of 13,980 valid data. Literature is downloaded after the screening in the format of "summary, full record (including references)," saved as plain text files used for analyzing data samples, and finally imported to Citespace, Scimago Graphica and other visual software. By combining the documentary metrology analysis, visualization analysis and social network analysis from three dimensions of the total amounts, citations and themes, we conclude the basic profiles and development dynamics of gene editing research.

Supplementary Table S1 Search strategy for investigating gene editing in the Web of Science (updated January. 19, 2022)

Component	Search Strategy
Gene editing	TS=((Meganuclease OR "zinc finger nucleases*" OR "Zinc finger endonuclease*" OR "zinc-finger nucleus" OR "TALE nuclease" OR "TALEN" OR TALN OR TALENS OR TALNS OR "Transcription activator-like effector*" OR "Transcription activator-like nu-clease*" OR "TAL effector nucleases" OR "TAL nuclease*" OR "clustered regularly in-terspaced short palindromic repeat*" OR "CRISPR*" OR "RNA-Guided Cas9" OR "ef-fector nucleases" OR "base editing" OR "Multiplex gene editing" OR "Cas9-guide RNA ribonucleoprotein " OR RNP OR "prime editing guide RNA" OR pegRNA OR "Prime editors" OR PE OR "genome editing" OR "gene editing" OR "engineered endonucle-ase*" OR "Engineered nuclease*" OR "site-directed nuclease*" OR "genome engineering" OR "chimeric nucleases" OR "Targeted modification" OR "Sequence-Specific Nucleases") AND ((Genome OR Gene OR Genetic) NEAR/2 Editing))
Meganuclease	TS= Meganuclease
ZFNs	TS=("zinc finger nucleases*" OR "Zinc finger endonuclease*" OR "zinc-finger nucleus")
TALENs	TS=("TALE nuclease" OR "TALEN" OR TALN OR TALENS OR TALNS OR "Transcription activator-like effector*" OR "Transcription activator-like nu-clease*" OR "TAL effector nucleases" OR "TAL nuclease*")
CRISPR-Cas	TS=("clustered regularly in-terspaced short palindromic repeat*" OR "CRISPR*" OR "RNA-Guided Cas9" OR "base editing" OR "Multiplex gene editing" OR "Cas9-guide RNA ribonucleoprotein " OR RNP OR "prime editing guide RNA" OR pegRNA OR "Prime editors" OR PE)
Regulatory	TS=(Regulat* OR Legistlat*) and ((Meganuclease OR "zinc finger nucleases*" OR "Zinc finger endonuclease*" OR "zinc-finger nucleus" OR "TALE nuclease" OR "TALEN" OR TALN OR TALENS OR TALNS OR "Transcription activator-like effector*" OR "Transcription activator-like nu-clease*" OR "TAL effector nucleases" OR "TAL nuclease*" OR "clustered regularly in-terspaced short palindromic repeat*" OR "CRISPR*" OR "RNA-Guided Cas9" OR "ef-fector nucleases" OR "base editing" OR "Multiplex gene editing" OR "Cas9-guide RNA ribonucleoprotein " OR RNP OR "prime editing guide RNA" OR pegRNA OR "Prime editors" OR PE OR "genome editing" OR "gene editing" OR "engineered endonucle-ase*" OR "Engineered nuclease*" OR "site-directed nuclease*" OR "genome engineering" OR "chimeric nucleases" OR "Targeted modification" OR "Sequence-Specific Nucleases") AND ((Genome OR Gene OR Genetic) NEAR/2 Editing))

TS =Title, Abstract and Keywords; Indexes=SCI-EXPANDED

Supplementary Table S2 Gene editing-related records

No	Document type	Records	No	Document type	Records
1	Article	9983	10	Corrections	90
2	Review	3997	11	Book Reviews	20
3	Meeting Abstract	1614	12	Data Papers	3
4	Editorial Materials	762	13	Retracted Publications	2
5	Book Chapters	266	14	Bibliographies	1
6	Proceedings Papers	190	15	Biographical-Items	1
7	Letters	180	16	Expression Of Concern	1
8	News Items	155	17	Publication With Expression Of Concern	1
9	Early Access	123	18	Retractions	1

Note: Our analysis focuses on published articles and reviews only.

Supplementary Table S3 Sub-technologies and Sub-sectors publishing on gene editing since 2000

	Sub-technologies				Sub-sectors		
Years	CRISPR/Cas	ZFNs	TALENs	Meganuclease	Medicine	Industry	Agriculture
2000	0	0	0	0	0	0	0
2001	0	0	0	0	1	0	0
2002	0	0	0	0	0	0	0
2003	0	0	0	0	0	0	0
2004	0	0	0	0	0	0	0
2005	0	2	0	0	5	0	0
2006	0	0	0	0	3	0	0
2007	0	3	0	0	4	0	0
2008	0	3	0	0	2	0	0
2009	0	4	0	0	6	0	0
2010	0	8	0	0	2	1	0
2011	0	31	7	0	20	1	0
2012	1	34	18	1	36	6	1
2013	45	74	56	2	80	11	1
2014	201	168	171	4	223	17	4
2015	375	162	146	1	341	74	14
2016	691	194	206	6	575	145	37
2017	958	154	151	4	751	208	59
2018	1175	161	163	4	979	295	84
2019	1408	93	114	3	1075	438	134
2020	1547	94	121	4	1240	534	135
2021	1601	82	114	7	1333	627	164
total	8002	1267	1267	36	6676	2357	633

Supplementary Table S4 Leading countries publishing (GE)

Countries	Number of records	Total citations	Average citations
USA	5926	277961	46.91
China	3308	87818	26.55
Japan	1144	26281	22.97
Germany	1071	32795	30.62
UK	1015	29839	29.40
South Korea	558	17460	31.29
France	541	16848	31.14
Canada	528	12122	22.96
Australia	526	10785	20.50
India	491	6408	13.05
Netherlands	444	15737	35.44
Italy	399	12674	31.76
Spain	332	8870	26.72
Switzerland	282	8978	31.84
Denmark	233	7047	30.24
Scotland	212	4734	22.33
Sweden	208	17530	84.28
Russia	195	4345	22.28
Belgium	164	3127	19.07
Iran	152	1600	10.53

Supplementary Table S5 Frequency of GE keyword occurrence (Top 100)

No.	Key Words	Frequency	No.	Key Words	Frequency
1	CRISPR/Cas9	2606	51	efficient	270
2	expression	1874	52	cancer	265
3	gene	1591	53	crystal structure	259
4	DNA	1217	54	RNA guided endonuclease	259
5	genome	1184	55	transformation	256
6	mutation	1021	56	growth	254
7	RNA	963	57	transcription factor	248
8	protein	918	58	CRISPR	247
9	cell	850	59	mammalian cell	245
10	gene therapy	776	60	genomic DNA	244
11	generation	749	61	T cell	238
12	in vivo	696	62	recombination	234
13	nuclease	658	63	inhibition	229
14	Zinc Finger Nuclease	643	64	vector	221
15	homologous recombination	634	65	cpf1	216
16	endonuclease	617	66	reveal	216
17	specificity	611	67	efficiency	208
18	gene expression	608	68	messenger RNA	207
19	identification	605	69	design	206
20	human cell	541	70	binding	201
21	targeted mutagenesis	515	71	base	199
22	mouse model	514	72	integration	194
23	mice	512	73	knockout	191
24	pluripotent stem cell	511	74	TALEN	190
25	activation	477	75	recognition	187
26	stem cell	469	76	embryonic stem cell	178
27	in vitro	462	77	target	177
28	resistance	456	78	saccharomyces cerevisiae	171
29	mechanism	425	79	receptor	166
30	double strand break	380	80	synthetic biology	165
31	delivery	377	81	tool	161
32	mutagenesis	371	82	infection	159
33	arabidopsis	370	83	arabidopsis thaliana	156
34	therapy	351	84	transplantation	155
35	transcription	350	85	genome engineering	154
36	plant	346	86	lentiviral vector	149
37	guide RNA	344	87	induction	147
38	disease	343	88	gene knockout	145

39	differentiation	343	89	immune system	142
40	sequence	335	90	replication	141
41	model	333	91	homology directed repair	140
42	cleavage	330	92	genetic engineering	139
43	escherichia coli	327	93	biosynthesis	139
44	one step generation	313	94	disruption	139
45	evolution	310	95	deletion	135
46	repair	308	96	induced pluripotent stem cell	132
47	mouse	290	97	DNA cleavage	132
48	rice	288	98	diversity	130
49	complex	287	99	CRISPR Cas9 Nuclease	130
50	pathway	270	100	zebrafish	128

Supplementary Table S6 Number of publications (Total GE and GE regulatory)

	Number of total GE publications	Number of GE regulatory publications
1999	1	0
2000	2	0
2001	1	0
2002	0	0
2003	1	0
2004	1	0
2005	9	0
2006	6	3
2007	6	1
2008	7	1
2009	8	1
2010	10	2
2011	41	9
2012	60	10
2013	147	31
2014	429	100
2015	669	184
2016	1133	297
2017	1509	455
2018	1926	600
2019	2320	785
2020	2666	850
2021	2966	911

Supplementary Table S7 Frequency of GE regulatory keyword occurrence (Top 90)

No.	Key Words	Frequency	No.	Key Words	Frequency
1	expression	638	46	efficient	79
2	genome editing	553	47	cancer	78
3	gene	481	48	crystal structure	78
4	gene editing	318	49	RNA guided endonuclease	77
5	protein	294	50	transformation	77
6	gene expression	283	51	growth	75
7	genome	263	52	transcription factor	73
8	system	262	53	CRISPR	72
9	cell	246	54	mammalian cell	71
10	mutation	242	55	genomic DNA	71
11	dna	230	56	T cell	68
12	rna	228	57	recombination	68
13	identification	209	58	inhibition	67
14	activation	207	59	vector	65
15	in vivo	178	60	cpf1	63
16	cas9	174	61	reveal	60
17	transcription	173	62	efficiency	59
18	crispr cas9	159	63	messenger RNA	59
19	targeted mutagenesis	152	64	design	59
20	transcription factor	138	65	binding	57
21	differentiation	136	66	base	56
22	gene therapy	134	67	integration	56
23	generation	131	68	knockout	56
24	mechanism	124	69	TALEN	55
25	arabidopsis	122	70	recognition	54
26	mouse model	119	71	embryonic stem cell	51
27	endonuclease	115	72	target	51
28	stem cell	115	73	saccharomyces cerevisiae	51
29	nuclease	111	74	receptor	50
30	resistance	111	75	synthetic biology	50
31	human cell	111	76	tool	49
32	mice	109	77	infection	49
33	homologous recombination	108	78	arabidopsis thaliana	48
34	specificity	108	79	transplantation	48
35	growth	107	80	genome engineering	47
36	in vitro	103	81	lentiviral vector	47
37	zinc finger nuclease	102	82	induction	47

38	pathway	95	83	gene knockout	46
39	crispr/cas9	94	84	immune system	46
40	plant	93	85	replication	45
41	complex	93	86	homology directed repair	45
42	pluripotent stem cell	92	87	genetic engineering	44
43	escherichia coli	87	88	biosynthesis	44
44	inhibition	85	89	disruption	44
45	double strand break	84	90	deletion	41

Supplementary Table S8 National cooperation in GE regulatory research

No.	Countries	Count	No.	Countries	Count
1	USA	1567	22	Israel	41
2	China	906	23	Iran	38
3	Germany	304	24	Poland	33
4	Japan	298	25	Norway	32
5	England	277	26	Turkey	31
6	Australia	161	27	Brazil	28
7	India	154	28	Saudi Arabia	28
8	France	150	29	New Zealand	27
9	Canada	144	30	Finland	27
10	Netherlands	126	31	Taiwan	27
11	South Korea	120	32	South Africa	27
12	Italy	114	33	Egypt	25
13	Spain	98	34	Singapore	25
14	Switzerland	86	35	Czech Republic	23
15	Sweden	80	36	Ireland	22
16	Scotland	67	37	Argentina	21
17	Russia	59	38	Mexico	20
18	Belgium	57	39	Portugal	19
19	Pakistan	52	40	Malaysia	17
20	Austria	48	41	Hungary	15
21	Denmark	45	42	Greece	14
